# Evaluation of Graphite Nanoplatelets Influence on the Lubrication Properties of Asphalt Binders

**DOI:** 10.3390/ma13030772

**Published:** 2020-02-07

**Authors:** Tianhao Yan, Lorenzo Paolo Ingrassia, Ravi Kumar, Mugurel Turos, Francesco Canestrari, Xiaohu Lu, Mihai Marasteanu

**Affiliations:** 1 Department of Civil, Environmental and Geo- Engineering, University of Minnesota, Twin Cities, 500 Pillsbury Drive SE, Minneapolis, MN 55455, USA; yan00004@umn.edu (T.Y.); kumar432@umn.edu (R.K.); turos001@umn.edu (M.T.); 2 Department of Civil and Building Engineering and Architecture (DICEA), Università Politecnica delle Marche, Via Brecce Bianche, 60131 Ancona, Italy; l.p.ingrassia@pm.univpm.it; 3 Nynas AB, SE-14982 Nynäshamn, Sweden; xiaohu.lu@nynas.com

**Keywords:** graphite nanoplatelets (GNPs), asphalt binder, compaction, viscosity, tribology, lubrication, nanomaterials

## Abstract

With the major advance in nanotechnology, there has been an emerging interest in applying nanoscale materials to asphalt pavement materials. Among them, considerable interest has been directed to carbon-based nanomaterials, such as carbon nanotubes (CNTs) and graphite nanoplatelets (GNPs). Recent studies have proven that the addition of small percentages of GNPs could significantly reduce the compaction effort required to densify HMA. Viscosity measurements showed, however, that the addition of GNPs increased the viscosity of the binder. This observation pointed towards the presence of a different mechanism responsible for the reduction of compaction effort. A new test method used for lubricants and based on tribology has been recently proposed in order to characterize the lubricating behaviour of asphalt binders. In this study, the tribological characterization of an asphalt binder modified with GNPs was performed. A novel approach in which aggregate surface microtexture was simulated using rough surfaces of the testing fixtures, shows that indeed, the addition of GNPs lowers the friction coefficient and therefore, enhances the lubrication properties of the binder when mixed with mineral aggregates.

## 1. Introduction

Significant efforts have been devoted to developing new types of construction materials, which exhibit better mechanical performance and enhanced durability. With the major advance in nanotechnology, there has been an emerging interest in applying nanoscale materials to construction materials [1], including asphalt [2,3,4]. Among them, considerable interest has been directed to carbon-based nanomaterials. One common type of such materials is carbon nanotubes (CNTs). It was shown that the addition of CNTs could reduce fatigue and permanent deformation of asphalt mixtures, enhance the resistance to thermal cracking, and reduce aging [5,6,7]. However, the high cost of CNTs (which may be in the order of magnitude of 100 €/kg) makes them unsuitable for large-scale application to asphalt pavements. A much more cost-effective carbon-based nanomaterial is represented by graphite nanoplatelets (GNPs). The GNPs are nano-discs with a sub-micrometer diameter and a thickness of approximately one nanometer, produced from either graphene or natural graphite. If GNPs are prepared directly from graphene, each platelet typically consists of several layers of graphene sheets, which are a single layer of carbon atoms. Depending on its type and carbon purity, the cost of GNPs can be as low as 6 €/kg (i.e., about 3 $/lb), which is comparable to existing asphalt modifiers such as the styrene-butadiene-styrene (SBS) polymer, significantly lower than the cost of multi-wall CNTs. 

In recent studies, Le et al. [8,9] have shown that the addition of small amounts of GNPs to asphalt binders can significantly improve the cracking resistance of asphalt binders and mixtures at low temperatures. A moderate addition of GNPs of 3% to 6% by weight of the binder resulted in 130% increase in flexural strength. For some asphalt mixtures, the addition of 6% GNP by weight of binder almost doubled the fracture energy. However, one of the most interesting results was the significant reduction in the number of gyrations required to achieve a target air void content.

Viscosity measurements indicated, however, that the addition of GNPs increased the viscosity of the binder [10]. Such discrepancies between the binder viscosity and the mixture compaction behaviour have been already observed by other authors, who pointed out the drawbacks of an experimental approach based only on the study of viscosity [11,12]. In addition, other studies [13,14] have shown that the mixture compactability does not improve linearly with the temperature increase, but on the contrary, it gets worse above a certain level of temperature, although viscosity decreases progressively with temperature.

Most likely, a different mechanism is responsible for the reduction of compaction efforts. In this regard, a new test method, used for lubricants and based on tribology, has been recently proposed in order to characterize the lubricating behaviour of asphalt binders [15,16,17,18]. To date, this approach has been used mainly to investigate the lubricating properties of warm mix asphalt (WMA) binders, produced with different WMA technologies [17,19,20]. Other fields of application, such as the modification with nanomaterials, should be considered. Although the use of rough substrates in the tests is generally recommended for the study of asphalt binders [10,17], only smooth substrates have been used up to now in previous research.

Within this context, in this study, the tribological characterization of asphalt binders modified with GNPs was performed. A novel approach in which the aggregate surface was simulated using rough surfaces of the testing fixtures, shows that indeed the addition of GNPs lowers the friction coefficient, and, therefore, enhances the lubrication properties of the binder. 

## 2. Mechanism of Friction and Lubrication 

**Figure 1:**
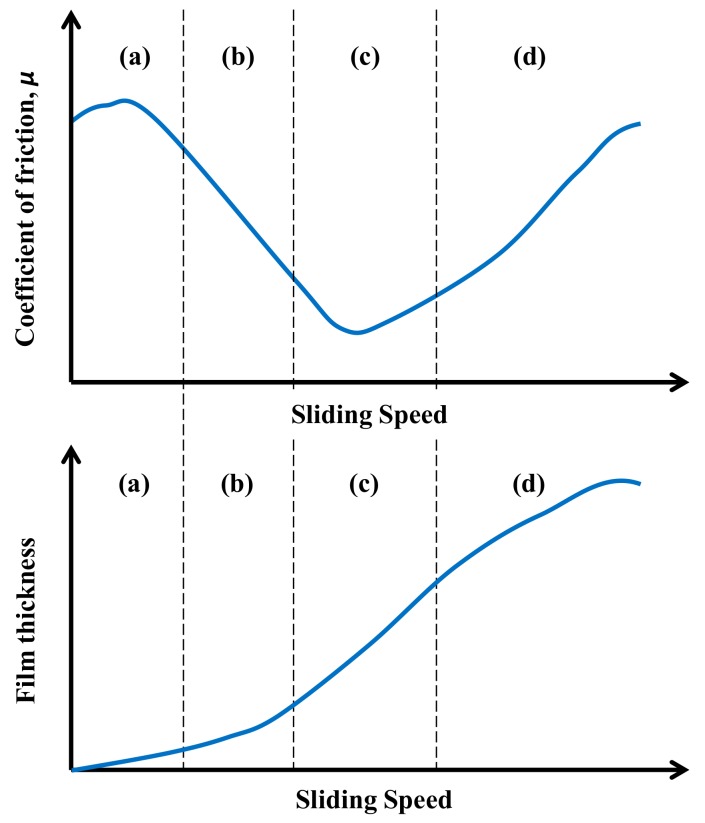
Stribeck curve.

In tribology, the lubrication properties of a material placed between two solids in relative motion is normally described through the Stribeck curve (Figure 1), which shows the evolution of the coefficient of friction *μ* as a function of the sliding speed [16,17]. The change in the coefficient of friction values is due to the variation of the thickness of the lubricating film, as shown in Figure 1. The Stribeck curve can be generally divided into four regions, which correspond to different regimes of lubrication [16,17]:

- the *boundary regime* (a), occurring when the lubricating film is thin and, consequently, a high *μ* is determined by the strong interaction between the asperities of the solids;
- the *mixed regime* (b), where a reduction of *μ* occurs, because of the increased thickness of the lubricating film, which reduces the direct contact between the solids;
- the *elasto-hydrodynamic regime* (c), in which the minimum *μ* is reached, because the thickness of the lubricating film is able to completely separate the solid surfaces;
- the *hydrodynamic regime* (d), where the film is so thick that there is a new increase of *μ*, depending on the viscous drag of the lubricant.

However, in addition to the sliding speed, other important parameters also govern the phenomenon. Since friction is not an intrinsic property of the material but of the overall system, it strongly depends also on the nature, surface roughness and wear of the solids in contact [16,17]. Such factors are extremely crucial mainly when the lubricating film is not thick enough to separate all solid asperities. Furthermore, the thickness of the lubricating film depends also on the normal load between the solids and, for thermo-dependent materials such as bitumen, on the temperature, which controls the viscosity [17].

**Figure 2:**
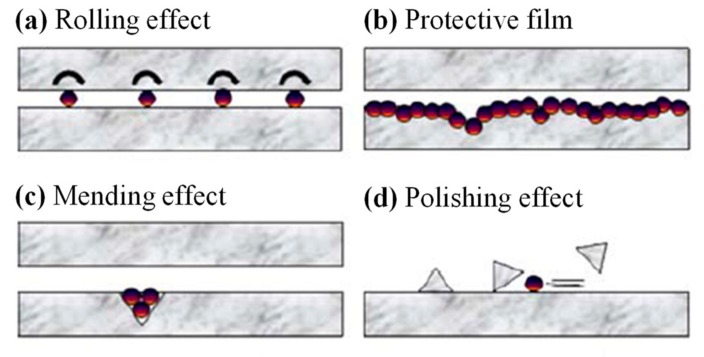
Lubrication mechanisms of nanoparticles (from [28]): (**a**) rolling effect; (**b**) protective film; (**c**) mending effect; (**d**) polishing effect.

Even though the use of nanoparticles to improve the lubrication properties of asphalt mix is new to the world of asphalt binder, their use in the lubrication industry is well established. The role of nanoparticles in friction reduction has been investigated by many researchers and the mechanisms involved can be described as follow: rolling effect [21,22], protective film [23,24,25], mending effect [26] and polishing effect [27]. The first two mechanisms belong to the direct effect of nanoparticles on lubrication improvement. Spherical nanoparticles are likely to roll between the frictional surfaces and play the role of ball bearings (Figure 2a). In addition, the nanoparticles form a thin protecting film on the surface thereby reducing the friction between two surfaces (Figure 2b). The other two mechanisms are the secondary effect of nanoparticles on surface enhancement. The nanoparticles deposit on the frictional surface forming a tribo-film to compensate for the loss of mass (mending effect, Figure 2c). In addition, the roughness of the rubbing surfaces is reduced due to the abrasiveness of the hard nanoparticles (polishing effect, Figure 2d).

**Figure 3:**
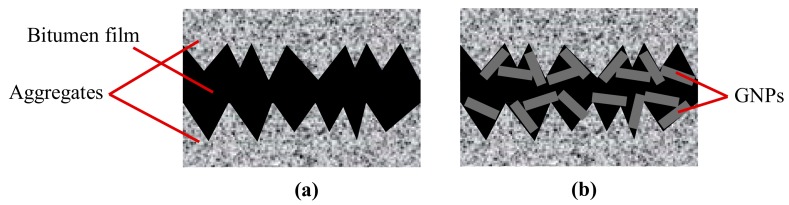
Scheme of the bitumen film between aggregate surfaces: (**a**) without GNPs; (**b**) with GNPs.

For the problem of interest (i.e., the compaction of GNP modified asphalt mixtures), a phenomenon similar to the mending effect is expected to occur, as hypothesised in a previous study [10]. Indeed, GNPs could place between the asperities of the aggregates, providing overall reduced roughness and thus enhanced lubrication with respect to the base bitumen, as schematized in Figure 3. 

## 3. Experimental Investigation

In this study, a plain PG58-28 bitumen was used as base binder. A GNP made of a synthetic graphite material with 99.66% carbon and 0.34% ash, characterized by an enhanced surface area equal to 250 m^2^/g, was added to the asphalt binder in two proportions: 3% and 6% by weight of the binder. The 3% and 6% blends were prepared at University of Minnesota (USA) using a high shear mixer. No clustering of GNPs was observed during the preparation of the samples. The blends were then stored in 85 g cans. Half of the cans were shipped to Nynas (Sweden) to be tested and the other half was kept and tested at University of Minnesota.

**Figure 4:**
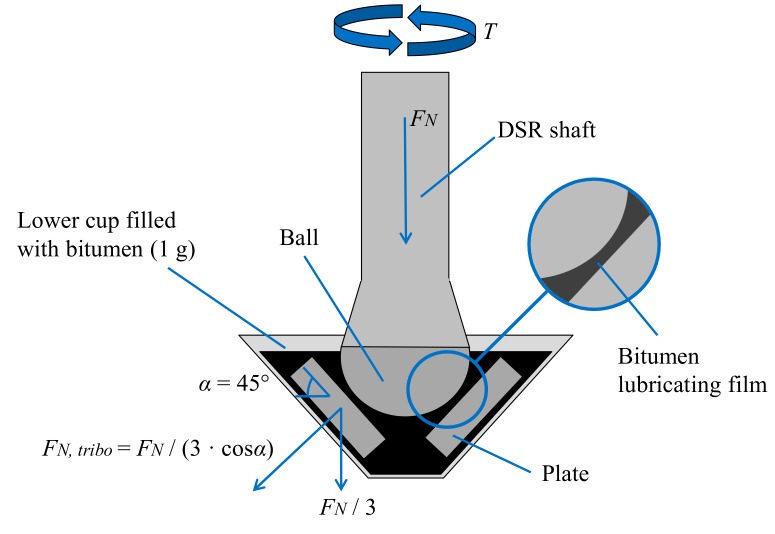
Scheme of the tribological fixture used for testing at Nynas.

Tribological tests were performed using a ball-on-three-plates fixture mounted on a Dynamic Shear Rheometer (DSR). The fixture employed at Nynas is schematized in Figure 4. 

The coefficient of friction *μ* is calculated as in Equation (1):

(1) $$\mu =\frac{F_{F-TOT}}{F_{N,\;tribo-TOT}}$$

in which *F_F-TOT_* and *F_N,__tribo-TOT_* are, respectively, the total friction force and the total normal force experienced by the specimen, determined according to Equations (2) and (3):

(2) $$F_{F-TOT}=3\cdot \left(\frac{T}{3\cdot r_{ball}\cdot \sin\alpha }\right)=\frac{T}{r_{ball}\cdot \sin\alpha }$$

(3) $$F_{N,\;tribo-TOT}=3\cdot \left(\frac{F_{N}}{3\cdot \cos\alpha }\right)=\frac{F_{N}}{\cos\alpha }$$

where *F_N_* is the DSR axial force, *T* is the torque, *r_ball_* is the radius of the ball and *α* is the angle between the plates and the horizontal plane (45° for the ball-on-three-plates fixture, see Figure 4). Since the fixture geometry is known, in order to calculate the coefficient of friction, it is sufficient to impose the axial force and the rotational speed and measure the resulting torque value.

**Figure 5:**
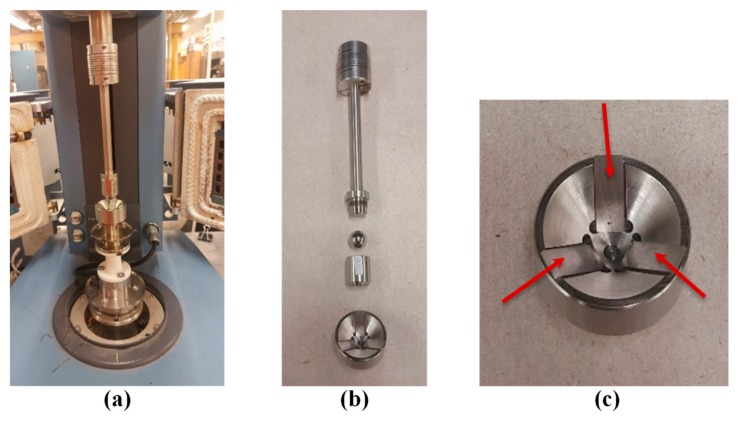
Tribological fixture used at University of Minnesota: (**a**) general view; (**b**) components of the fixture; (**c**) lower cup and testing plates.

The ball-on-three-plates fixture used at the University of Minnesota is similar to the one presented in Figure 4, but some parts are slightly different from the ones used at Nynas. As shown in Figure 5, the fixture has five different components: a lower cup, three steel plates, a steel ball, a shaft and a ring to attach the ball to the shaft. In the lower cup there are three plates with an angle of 45° with respect to the horizontal plane and the asphalt sample. The steel ball is attached to the shaft, which then gets attached to the DSR head. It is worth pointing out that, unlike the fixture used at Nynas, in which the plates are screwed and therefore perfectly fixed in the lower cup, in the fixture used in Minnesota the plates are not screwed but they are simply placed into three flat grooves in the lower cup (Figure 5c). For the abovementioned reason, they have a certain degree of freedom to move at the very beginning of the test, resulting in an initial compliance of the fixture not observed with the equipment used at Nynas, as shown hereafter. 

In order to simulate as much as possible the typical compaction temperatures for hot mix asphalt (HMA) and warm mix asphalt (WMA) mixtures, 110 °C, 130 °C and 150 °C were considered as testing temperatures. Steel ball and steel plates were used as substrate in all tests. Specifically, the contact points between ball and plates were always different for each specimen, in order to avoid the influence of wear, thus reducing the number of variables in the experiments. The axial force *F_N_* was kept constant and equal to 10 N during the tests, whereas the rotational speed was increased in logarithmic steps from 0.1 to 1433 rpm. These testing conditions were chosen for comparison with previous studies [15,17]. All tests were performed based on a protocol previously developed by Ingrassia et al. [17].

The experimental investigation also included viscosity tests. In Sweden, the tests were performed using the DSR cone and plate geometry, characterized by a radius of 20 mm and a slope of 2°, while in Minnesota viscosity was obtained with a Brookfield viscometer (Brookfield Engineering, Middleboro MA, USA). The same temperatures as tribological tests were investigated (i.e., 110 °C, 130 °C and 150 °C). At Nynas, the specimens were tested starting from the highest temperature and then the testing temperature was progressively reduced. At each temperature, a shear rate sweep was carried out by increasing the shear rate in logarithmic steps, with the aim of evaluating the Newtonian or non-Newtonian behaviour of the binders. The range of shear rates investigated varied depending on the testing temperature (lower shear rates at lower temperatures to limit the torque applied). A shear rate sweep, consisting in five viscosity measurements for each testing temperature, was performed also at the University of Minnesota with the Brookfield viscometer. All testing performed at Nynas was done using an Anton Paar DSR equipment (Anton Paar GmbH, Graz, Austria), while all tribological testing performed at University of Minnesota was done using an AR 2000 TA Instruments DSR equipment (TA Instruments, New Castle DE, USA).

## 4. Results

### 4.1. Viscosity Results

**Figure 6:**
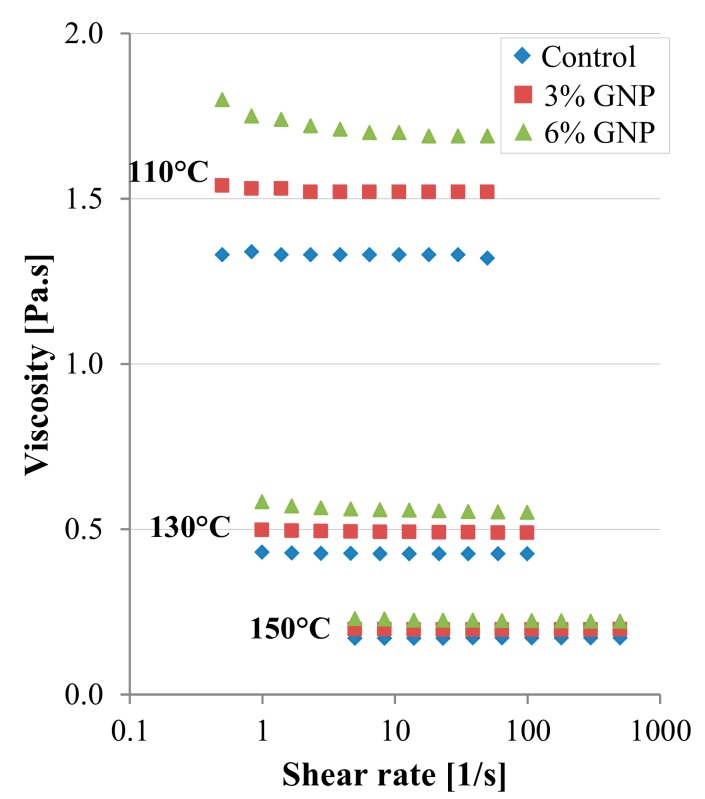
Cone and plate viscosity results (Nynas).

As an example, Figure 6 shows the viscosity results obtained at Nynas. As expected, the viscosity values decrease with the increase in temperature. Moreover, it is worth noting that the adopted cone and plate configuration seems reliable for evaluating the shear rate dependency of the binders. In this sense, only for the binder with 6% GNP at 110 °C the viscosity value may slightly depend on the shear rate.

Similar results were obtained at the University of Minnesota by using a Brookfield viscometer. Table 1 summarizes the average viscosity values at 110 °C, 130 °C and 150 °C, at which Newtonian behaviour could be broadly assumed for all binders. It can be noted that the viscosity values obtained at Nynas and University of Minnesota are generally comparable. Based on the values of the cone and plate viscosity, the increase in viscosity (with respect to the control bitumen) due to the addition of GNPs is approximately equal to 15% and 30% for the binders with 3% GNP and 6% GNP, respectively, at all testing temperatures. In the case of Brookfield data, the viscosity increase is smaller (around 5%–10% for the blend with 3% GNP and about 25% for the blend with 6% GNP).

In summary, these results confirm that the improved workability of GNP mixtures, observed by Le et al. [8,9], cannot be explained by a viscosity reduction.

### 4.2. Tribological Results Using Smooth Surfaces

**Figure 7:**
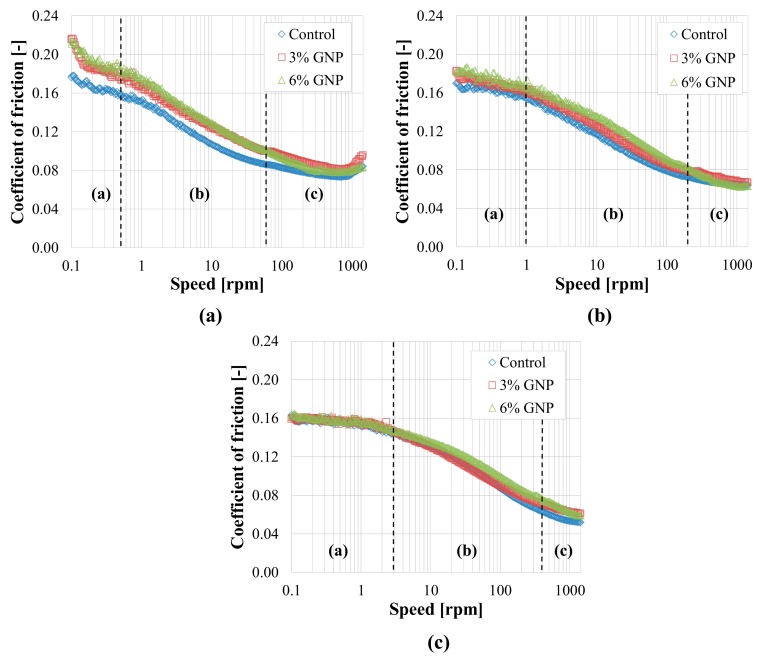
Tribological results with smooth surfaces obtained at Nynas: (**a**) 110 °C; (**b**) 130 °C; (**c**) 150 °C.

**Figure 8:**
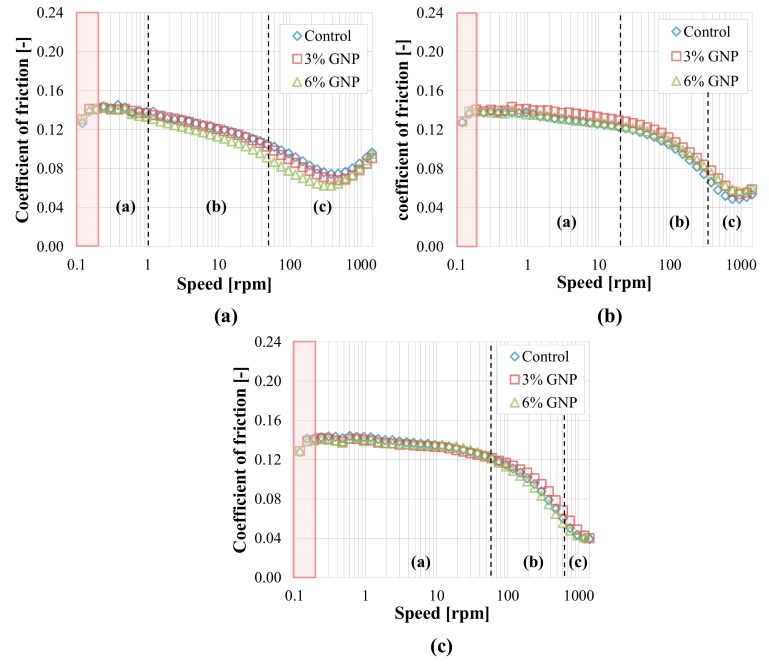
Tribological results with smooth surfaces obtained at University of Minnesota: (**a**) 110 °C; (**b**) 130 °C; (**c**) 150 °C.

The tribological results obtained with smooth substrates are shown in Figure 7 (Nynas) and Figure 8 (University of Minnesota). At Nynas, the Stribeck curves were obtained as the average of at least eight replicates (four consecutive replicates on each specimen tested). Specifically, according to the protocol by Ingrassia et al. [17], the first replicate on the specimen was discarded, because it was considered as a “pre-run” to allow the formation of the lubricating film. At University of Minnesota, the Stribeck curves were obtained similarly, by discarding the first replicate and considering the average of the five subsequent replicates on the specimen.

From Figure 7a, at 110 °C, the boundary (a), mixed (b) and elasto-hydrodynamic (c) regimes can be observed at very low, intermediate and high speeds, respectively. With the temperature increase, the same regimes of lubrication are identified for progressively higher values of sliding speed (Figure 7b,c), due to the decreased viscosity of the binder. Moreover, a general lubrication improvement is achieved as the temperature increases: in fact, the values of *μ* are between 0.08 and 0.22 at 110 °C, between 0.06 and 0.18 at 130 °C and between 0.05 and 0.16 at 150 °C (Figure 7). As for the results from University of Minnesota (Figure 8), the same regimes are observed as well as their shift towards higher speeds when temperature increases. However, the intervals of speed related to every lubrication regime are relatively different as compared to Nynas results. In addition, in general, lower values of *μ* were obtained in Minnesota with respect to those obtained in Sweden, probably because of the slightly different properties of the ball and plates provided by the two manufacturers (and, more generally, due to the differences between the devices employed). Nevertheless, the determination of the absolute value of the friction coefficient is not the main focus of this study, whose objective is primarily to evaluate the effect of GNP modification (which is discussed below). It has also to be noted that the main difference between the values of *μ* measured at Nynas and those measured at University of Minnesota is at very low speeds (≤0.2 rpm), in the region highlighted in Figure 8. As already explained above, this difference is due to the fact that the fixture employed in Minnesota shows a certain compliance at the beginning of the test because of the sliding of the plates. Consequently, these results should be neglected. Even in this case (as for the Nynas results), a slight reduction of friction is observed as temperature increases, especially in the elasto-hydrodynamic regime (c) (Figure 8). 

As far as the comparison between the binders is concerned, a general increase of the coefficient of friction can be noticed for all temperatures and lubrication regimes after adding the GNPs in the case of Nynas results (Figure 7). Specifically, the blend with 3% GNP generally exhibits intermediate values of *μ* as compared to the control bitumen and the blend with 6% GNP. On the contrary, in terms of ranking of the binders, a clear trend linked to GNP content cannot be observed from the University of Minnesota results (Figure 8). It should be also noted that, in general, the differences between the blends seem smaller with the increase in temperature (Figure 7 and Figure 8).

Despite the differences between the results obtained in Sweden and those obtained in Minnesota, it can be stated that, overall, the proposed test method (adapted from the lubricants’ field) is appropriate to investigate the lubricating properties of asphalt binders, with or without any kind of modification. Indeed, the theoretical lubrication regimes (see Figure 1) can be qualitatively identified from the analysis of the Stribeck curves.

However, in both cases (Figure 7 and Figure 8), the possible improvement of the lubrication properties of the control binder due to the addition of GNPs is not observed, at least for the testing conditions considered. 

**Figure 9:**
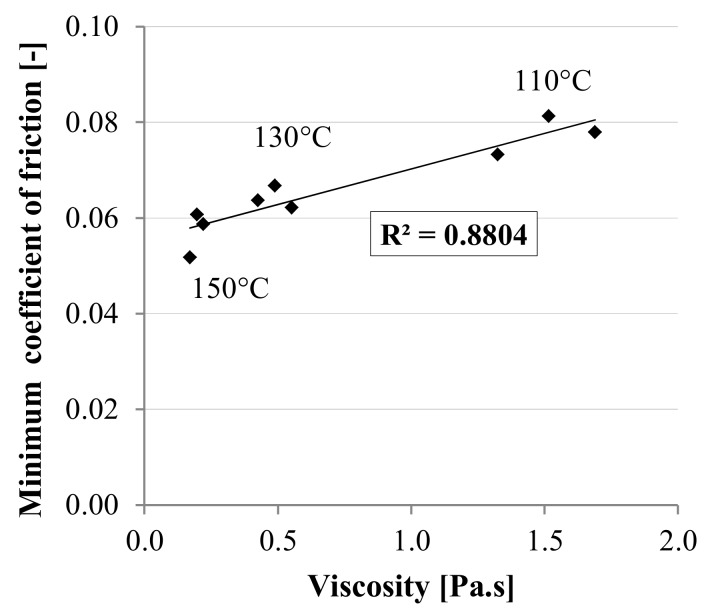
Correlation between minimum coefficient of friction and viscosity, based on Nynas results.

As a possible interpretation of these results, Figure 9 shows a direct correlation between the viscosity and the minimum coefficient of friction measured in the elasto-hydrodynamic regime (c) for all binders and temperatures, based on the results obtained at Nynas. The high value of the correlation coefficient *R^2^* suggests that, in the elasto-hydrodynamic regime (c), the lubricating behaviour of the material mainly depends on its viscosity, as already observed by Ingrassia et al. [17] in a previous study on WMA binders. Therefore, given the increased viscosity obtained after the addition of GNPs, the potential lubrication improvement is not expected in this regime. In addition, it is unlikely that during the compaction of the asphalt mixture a thick film of bitumen completely separates the aggregates (as it would happen in the hydrodynamic regime (d)), due to the high working temperatures normally adopted. Consequently, a possible friction reduction due to GNPs should be sought in the boundary (a) and mixed (b) regimes, in which, however, the influence of the substrate properties is crucial.

Based on such considerations, a second testing phase was carried out, as described in the following section.

### 4.3. Tribological Results Using Rough Surfaces

**Figure 10:**
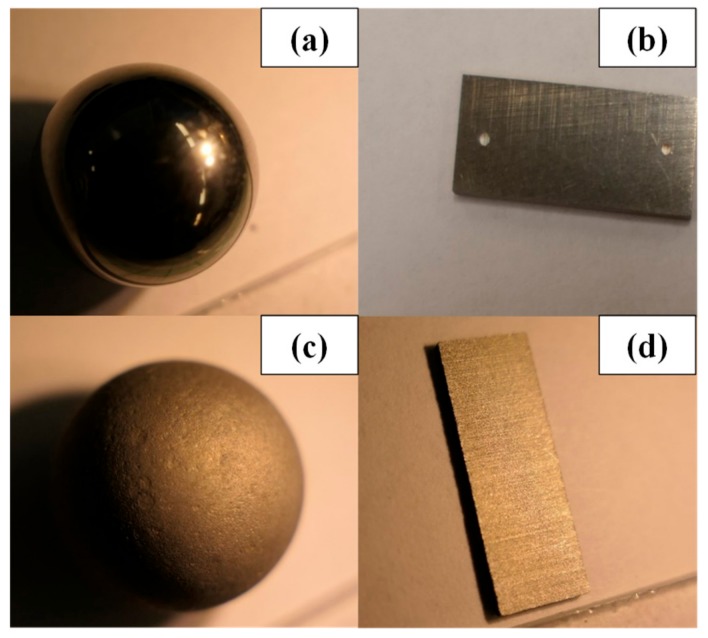
(**a**) smooth ball; (**b**) smooth plate (used); (**c**) rough ball; (**d**) rough plate.

During the first part of this study, the original manufactured geometry was used on both DSR devices, in which the ball and the plates have shiny and smooth surfaces. However, the use of smooth surfaces is not representative of the surface roughness of natural aggregates in asphalt mixtures. In the second testing phase, the surfaces of the ball and of the plates were roughened to better simulate the surface of the aggregates. The method consisted in immersing the ball and the plates in hydrochloric acid (HCl) for three days. Hydrochloric acid corroded the surfaces of the parts and made them rough and looking like an orange skin. Figure 10a,b present the original smooth ball and plate, whereas Figure 10c,d present the ball and plate after they were removed from the acid.

Even for these tests, the contact points between ball and plates were always different for each specimen to avoid the influence of wear. During all the tests, the axial force was kept constant and equal to 10 ± 0.1 N, while the rotational speed was increased in logarithmic steps from 0.01 radian/sec (≈0.1 rpm) to 150 radian/sec (≈1433 rpm), analogously to the previous experimental phase. All tests were carried out at University of Minnesota.

**Figure 11:**
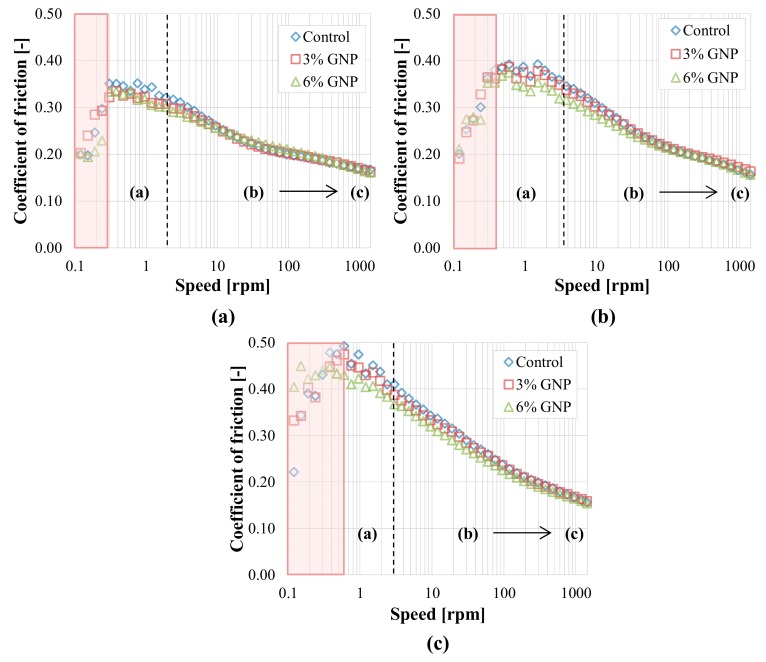
Tribological results with rough surfaces (University of Minnesota): (**a**) 110 °C; (**b**) 130 °C; (**c**) 150 °C.

The test performed using the ball and three plates with rough surfaces resulted in a different output. The adoption of rough surfaces (Figure 11) implies—as somehow expected—a significant increment of the coefficient of friction, which is up to three times higher (even more at 150 °C) as compared to the case of smooth substrates (Figure 8). A change in the lubrication regimes can be also noted with respect to Figure 8, as the boundary (a) and mixed (b) regions are identified at all temperatures, but the distinction between them is not very clear from the shape of the curves (Figure 11). In addition, at all temperatures, the minimum of the Stribeck curve is not reached for any binder, probably because under such conditions the complete separation between the solid asperities is harder to be achieved and the minimum shifts towards higher speeds, even though the mixed (b) regime tends to the elasto-hydrodynamic (c) one at high speeds. As already noticed for the smooth substrate (Figure 8), also in this case the values of *μ* measured at low speeds should be neglected, due to the sliding of the plates in the lower cup. For the rough surfaces, however, the initial value of *μ* at low speeds is around 0.2 for all binders and temperatures (Figure 11), whereas for the smooth substrate this value was around 0.12 (Figure 8). Such different values somehow provide a measure of the friction given by the steel-on-steel contact, which is obviously higher for the rough surfaces and almost independent from the testing temperature. Moreover, the speed range where the possible sliding of the plates occurs gets wider with the temperature increase (up to about 0.3 rpm at 110 °C, 0.4 rpm at 130 °C and 0.6 rpm at 150 °C, as highlighted in Figure 11). It is interesting to observe that, contrarily to what emerged in the case of smooth substrates (Figure 7 and Figure 8), the coefficient of friction remarkably increases for all binders as the testing temperature is increased, especially in the boundary regime (a). This is probably due to the fact that the decrease of the binder viscosity allows a greater contact between the solid asperities, resulting in higher friction. This finding suggests that a temperature increase may not always be beneficial for the compaction of the asphalt mixture.

As for the effect of GNP modification, at all three temperatures the coefficient of friction is reduced by the addition of GNPs in the boundary (a) as well as in the mixed (b) regime (Figure 11). At all temperatures, the lubrication properties of the binder are progressively improved as the GNP amount increases. Specifically, the friction reduction with respect to the control bitumen increases with the temperature and it is about 20% for the blend with 6% GNP at 150 °C. Conversely, the results are almost the same for all blends once the speed increases and the regime tends to the elasto-hydrodynamic (c) one. 

These results provide a possible explanation for the reduced compaction effort required to densify HMA with GNPs observed in previous studies [8,9]. During the compaction process, conditions comparable to the boundary (a) and mixed (b) ones may occur, and, thanks to an effect similar to the mending one, the nanoparticles stored on the aggregates’ rough surface may improve the compaction properties of asphalt mixtures.

## 5. Conclusions

The objective of this study was to examine the effect of graphite nanoplatelets (GNPs) on the lubricating behaviour of asphalt binders in an attempt to correlate the lubrication properties of the GNP modified binder with the enhanced compactability observed for GNP modified asphalt mixtures. Three binders were tested: the PG 58-28 binder (control), the PG 58-28 binder with 3% of GNP by weight, and the PG 58-28 with 6% of GNP by weight. Both viscosity and tribological tests were conducted to study the viscous and lubricating behaviour of the binders, respectively. In the tribological experiments, smooth and rough substrates were considered.

The main outcomes of the study can be summarized as follows:

- the viscosity of the binder increases with the quantity of GNPs, confirming that the reduced compaction efforts for GNP asphalt mixtures cannot be attributed to the reduction in the viscosity of the binder;
- GNPs do not improve the lubricating behaviour of the binder in the case of smooth substrates. Conversely, when rough substrates are considered, the lubrication properties of the binder are progressively improved in the boundary and mixed regimes as the GNP amount increases;
- since the rough substrate mirrors the actual aggregate roughness more accurately than the smooth substrate, the enhanced workability of GNP modified mixtures can be attributed to the fact that GNPs may occupy the space between the asperities of the aggregates, reducing the overall roughness and thus improving the lubrication;
- the tribological tests performed with rough substrates demonstrate that, for a given binder, friction increases significantly as the temperature increases (i.e., the viscosity decreases), especially in the boundary regime. This finding once again confirms that the viscosity is not the only parameter involved in the compaction of asphalt mixtures, as the interaction between the aggregates plays a crucial role.

Future work should be aimed at investigating in more details the correlation between compaction data and tribological data, assessing more asphalt binders and modifiers with the proposed approach, and identifying a representative tribological parameter. 

## Figures and Tables

**Table 1:** Average viscosity values of the binders tested (Pa.s).

Temperature (°C)	Control (PG58-28)		3% GNP		6% GNP	
	Cone & Plate	Brookfield	Cone & Plate	Brookfield	Cone & Plate	Brookfield
110	1.32	1.28	1.52	1.35	1.69	1.57
130	0.43	0.37	0.49	0.40	0.55	0.48
150	0.17	0.15	0.20	0.16	0.22	0.19
